# The Turing Teacher: Identifying core attributes for AI learning in K-12

**DOI:** 10.3389/frai.2022.1031450

**Published:** 2022-12-14

**Authors:** Alexander Pelaez, Amal Jacobson, Kara Trias, Elaine Winston

**Affiliations:** ^1^Information Systems and Business Analytics, Hofstra University, Hempstead, NY, United States; ^2^Progressive School of Long Island, Merrick, NY, United States; ^3^5E Analytics LLC, Merrick, NY, United States

**Keywords:** artificial intelligence (AI), education, K-12, executive function, efficacy

## Abstract

**Introduction:**

Artificial intelligence in the educational domain has many uses; however, using AI specifically to enhance education and teaching in a K-12 environment poses the most significant challenges to its use. Beyond usage and application, the quality of the education is made even more arduous due to the dynamics of teaching primary and secondary school children, whose needs far exceed mere fact recollection. Utilizing prior research using AI in education and online education in the K-12 space, we explore some of the hurdles that AI applications face in K-12 teaching and provide core attributes for a “Turing Teacher,” i.e., an AI powered technology for learning, specifically targeting the K-12 space.

**Methods:**

Using a survey, which included qualitative responses during the implementation of online learning during the Covid Pandemic, we analyze the results using univariate and multivariate tests and analyzed the qualitative responses to create core attributes needed for AI powered teaching technology.

**Results:**

The results present the challenges faced by any technology in an education setting and show that AI technology must help overcome negative feelings about technology in education. Further, the core attributes identified in the research must be addressed from the three stakeholder perspectives of teachers, parents and students.

**Discussion:**

We present our findings and lay the groundwork for future research in the area of AI powered education. The Turing Teacher must be able to adapt and collaborate with real teachers and address the varying needs of students. In addition, we explore the use of AI technology as a means to close the digital divide in traditionally disadvantaged communities.

## Introduction

The phrase “artificial intelligence” evokes visions pulled straight from science fiction films. Scenes of AI technologies interfacing with students from screens or intelligent robots acting as teachers, replacing the traditional teacher would reverberate through the imagination of any educator. One of the first examples of an actual AI robot teacher was “Ms. Brainmocker” from the television show the Jetsons, which aired in 1963 (Smithsonian Institution, 2013). Is the vision of an AI teacher really that farfetched? Practically, there are many different uses of artificial intelligence in the AI space beyond teaching. Researchers have studied AI in administration, instruction and learning (Chen et al., 2020), in which the impact of these technologies are examined; however, the broad nature of technologies discussed in research, such as learning analytics, data mining, online learning, and student/teacher interfaces create a gap in understanding the application of these AI related technologies in education.

The Holy Grail for AI in education is how AI technologies can be used in learning. To understand how artificial intelligence can be effective at facilitating education, we first need to understand how learning occurs in normal human-to-human interactions. In 1950, Alan Turing proposed a test called the imitation game, which is considered the first test of artificial intelligence. The test proposes that for a successful system to be considered artificial intelligence, a human should be unable to distinguish a conversation with a human from a conversation with a system (Muggleton, 2014). The difficulties encountered to make a system indistinguishable from a real teacher are numerous, and involve dynamic interactions with students such as helping students organize and prioritize work, recognizing difficulties beyond subject matter, or even social issues like working in groups. Authors have advocated for more detailed review especially in the psychological concepts of “intelligence,” when discussing the “human intelligence” or “artificial intelligence” (Neubauer, 2021). The AI literature centers on topics like adaptive learning in a challenge-response method, but is limited in critical aspects of human-to-human interactions necessary for increased quality in learning. Our aim was to leverage data collected during the pandemic from a school forced into an online learning environment. We collected data from teachers and parents, and thus students by proxy, to identify perceptions and attitudes toward technology based learning, and we also delivered key themes or topics teachers and parents felt was essential for quality education. These concepts were then explored using the lens of AI in education to present core attributes that would be needed for effective AI technologies in K-12 learning.

## Literature in education technology

### Technology in education

The view of AI technologies has generally been to view the systems as a set of processes and their response, with a focus on autonomy, adaptability and interactivity (Dignum, 2021). These are core technological focus areas that researchers believe AI systems should possess. While autonomy, adaptability and interactivity are important, they may fail to capture some essential criteria as to what constitutes an effective education in the K-12 range, specifically skills human teachers empower in their students, such as self-efficacy, technical capabilities, and socialization skills. Samuel (2021) expands the notion of Dignum (2021), articulating the notion that AI technologies should mirror not only the actions of humans, but also the expressions of “human intelligence, cognition and logic.” This creates a need to expand the research in AI to define a clearer set of characteristics around how AI technologies should perform in order to achieve effective AI in education. We have at our disposal the unique circumstances to which the education space was subjected in the past few years brought about by the pandemic creating enormous challenges for all stakeholders, i.e., teachers, students and parents.

The same challenges faced by teachers in an online education environment would be exponentially magnified for artificial intelligence applications. Overcoming these challenges is no small task, as any teacher who has had to conduct remote teaching to an elementary school student in the last few years would attest. Studies find that online teaching becomes more effective when more socialization cues are added, and when key metrics such as executive functioning are integrated into its approach, all of which would also equally apply to developing an effective artificial intelligence system for education. Applying findings from studies conducted in an online learning environment would serve as a great foundation for understanding artificial intelligence in education.

Previous research in decision support systems have referenced the very same focus on how the technologies interacted with humans and the effect these systems had on both individual and group tasks. Various information systems have been used extensively to disseminate information within organizations for decades in the form of Knowledge Support Systems (KSS) and Decision Support Systems (DSS). The purpose of these systems was to provide the necessary information for decision making and information dissemination. DSS Design theory focused on the necessary calibration of the technology for the most effective use and outcomes (Kasper, 1996). Sankar et al. (1995), in the mid-1990s, discuss the need for adaptability in the interface in DSS systems and its effect on positive decision making. Increased interactivity also had a positive effect on better decision making (Gonzalez and Kasper, 1997). Finally, Ulfert et al. (2022), studied the effects of autonomy at various levels, i.e., high autonomy and lower autonomy of the DSS and found higher levels of autonomy in DSS led to lower levels of information overload, but may have a negative impact on technostress and intention to use.

Even the line between artificial intelligence and decision support systems may be more gray than black and white. López-Fernández et al. (2011) state that Knowledge Support Systems and Decision Support Systems are an extension of artificial intelligence research. Phillips-Wren (2012) discusses the use of AI algorithms as making these systems “intelligent” used across a number of domains such as finance and healthcare, which can be generated from a number of techniques such as Neural Networks and Machine Learning. Furthermore, as early as 1995, Turban noted these types of systems with intelligence as being Expert Decision Support Systems (Turban, 1995).

Therefore, while the foundations and goals of AI seem to be consistent, it isn't clear what would constitute a true AI enabled tool that would meet specific goals in the education space. Each domain itself may have different needs. For example, a finance AI may be able to work autonomously and have no interaction with a financial trader (Tadapaneni, 2019). In the healthcare space, the AI tool may serve as a true decision support system with a feedback loop from a healthcare provider, or be held in check by human interaction, such as what may occur in surgery (Panch et al., 2019). In education, though the interaction is very different, when discussing instruction or learning, the AI is interacting with a human at both a qualitative and quantitative level. This interaction is dynamic since the “target system” is not a financial transaction, which can be completely autonomous, or a diagnosis/treatment that can be derived from medical texts. Rather, the interaction is a constant feedback loop with the target being a human being at the K-12 level, one whose learning and responses are still being developed. Miller (2019) discusses the research being conducted in Human and AI interaction, specifically with robots, where the AI learns from human behavior who control the responses of the AI technology, thus creating a learning loop where “the AI learns from the human and the human learns from the AI.” Therefore, identifying the core attributes and applications of AI needs to be studied more carefully since the outcome and performance have a high degree of subjectivity due to the quality of the response.

In order to understand how to apply AI in educational settings, it is necessary to develop a foundational understanding of the challenges technologies create in a learning environment. Education has transformed significantly over the years and as more schools and educational organizations provide material in an online setting, leveraging the successes and failures of online learning should help in creating a strong paradigm for the development of AI in the education space.

### Online learning

There is a rich body of literature on technology based learning. Recently, especially due to the COVID-19 pandemic in 2020, the shift to online learning and the challenges associated with it are being reviewed and studied carefully. The term online learning is a more specific term for distance learning, which has been used since the first correspondence schools began, however, using the internet and internet related technologies create the foundation for online learning (Kentnor, 2015). Online learning seeks to convey information from a knowledge expert, a teacher, to a knowledge seeker, a student. The information systems literature is rich with how information is conveyed to users such as Media Richness Theory (Daft and Lengel, 1986) and Media Synchronicity Theory (Dennis et al., 2008) which together identify the relationship between the richness of information provided by an appropriate technology and the type of tasks being performed. The importance of having the correct technology to match with the type of tasks a user makes is essential. In an education setting, the conveyance and convergence tasks as defined by Dennis et al. (2008) represent the key functions of teachers and students, such as conveyance of information for knowledge and the convergence on a solution or answer by a student based on the information provided by the teacher.

The education field, however, in practice has had little time to adapt to the changing technologies and the actors within the field, i.e., the teachers and students have very different goals, measure outcomes differently, adapt differently with technology and respond to different technical stimuli differently. Education research into online learning has adapted to the changing needs from studying design issues and learning characteristics in the 1990's to exploring more complex interactions, such as communities of learning, more instructional design of classes, and innovation (Martin et al., 2020).

A significant amount of literature has focused on the online learning environments of adults, specifically college programs. Universities have more money than elementary and secondary schools for technology and training, thus making it easier to incorporate it into their existing offering. Entire markets have been made around online learning with universities that are exclusively online and the origination of MOOC's such as Coursera and Udemy (Wilson and Gruzd, 2014). Other environments for learning have also been created around coding, business classes, and even classes in art and music. Further, the platforms on which these classes are deployed, such as YouTube, are in many cases completely free of charge, as content creators are paid through advertising. The very broad and dynamic nature of these technologies makes the content unpredictable and less standardized, making the quality somewhat suspect for younger learners, as opposed to adult learners, who have a more mature filter for content (Neumann and Herodotou, 2020). Younger learners have a different set of objectives and needs than adults in college (Reed et al., 2022). Some of the key differences include the direct goal of attaining a job, learning a trade, or even a more dedicated desire to learn a subject. Younger learners have a completely different focus and do not have the same goal-oriented approach. Furthermore, beyond the basic skills of reading and writing, younger learners, in an online environment, begin to obtain critical skills needed for adapting to an ever changing business environment, such as interacting with others remotely and interfacing with technology for seeking knowledge (Roper, 2007; Kong et al., 2014). With a different set of goals and objectives and an even further disparity in learning methods and approaches, the AI for teaching K-12 must be more adaptive and sensitive to the needs of younger learners.

### Online learning in K-12 environments

The primary and secondary school system has not had many opportunities to develop online programs. Some K-12 programs do offer online education, mostly to a homeschooling market. The vast majority of school districts and private schools are simply not equipped to provide the necessary technological requirements, nor do they have the skill set to adequately navigate the technological challenges. IS research has shown that effective use of technology is predicated on technical efficacy and proficiency, which can be moderated by technical training. Ball and Levy (2008) found that computer self-efficacy was a significant predictor of intention to use technology, and thus those with higher self-efficacy would be more inclined not only to adopt the technology but be more effective at using the technology.

A number of other studies have shown the challenges of an online learning environment. Stress on teachers has a negative effect on a teacher's ability to conduct classes, and thus, has a negative effect on student performance (Oberle et al., 2020). Lower self-efficacy has been shown to lead to higher amounts of stress (Bandura, 1982) and the lower technological self-efficacy among teachers further increases negative emotions, leading to student's lower performance (Stephanou, 2011).

The stress of the online environment extends beyond the teachers and students to the parents. The negative perceptions parents may possess regarding online learning increases the overall stress, primarily due to the uncertainty of the pandemic, and compounded by an unfamiliar learning environment (Midcalf and Boatwright, 2020). Children absorb the opinions of the parents and project these negative opinions and emotions onto the online learning experience and become overwhelmed. The social regulation that occurs in a classroom among peers and teachers cannot be achieved in an online environment due to the absence of social cues. Sproull and Kiesler (1986) has shown that electronic communication reduces normal social cues that ordinarily regulate behavior. Children can become lost and wander, if not physically away from the screen, but emotionally and mentally, which cannot be regulated by a teacher or other students.

Finally, teachers' functions include feedback and support, which are difficult in an online asynchronous environment. Beyond providing information with a binary feedback system, i.e., correct/incorrect responses, children need feedback which can come in the form of comments, encouragement, and a positive tone (Mullikin, 2020). The impersonality of computer mediated communication makes this extremely difficult. The feedback loop is critical for students' learning and must be done in a manner and with vocabulary for the appropriate grade level. For university students it has been known that feedback can be provided in a manner which they feel is too late to be useful, too vague, unclear and inconsistent (Crook et al., 2012). The media type and synchronicity of the media have been found to impact the quality of the feedback. Written feedback has been shown to have issues due to handwriting legibility and complexity (Walker, 2009). Audio has some limitations as well but an enhanced form of feedback has shown to have a positive outcome (Nortcliffe and Middleton, 2008). Video feedback provides the most potential for positive feedback and has the potential to provide the most qualitative and interactive feedback of all the media (Abrahamson, 2010; Crook et al., 2012).

### Technology self-efficacy

The performance of AI, or AI empowered tools, in education cannot be studied in isolation. At its core, AI is a technology that will be interacting with key stakeholders, i.e., teachers and students, whether synchronous or asynchronous. Stakeholder interaction with the technology is a critical component and one of the core elements of this interaction is the technology self-efficacy of the stakeholder, thus, any lack of familiarity with a technology will create problems for its usage.

Self-efficacy is one's belief in their ability to execute a particular task or behavior (Bandura, 1982). Technology self-efficacy measures a user's confidence in the use of a particular technology (Compeau and Higgins, 1995). Research has shown that higher technology self-efficacy results in the belief that a successful outcome will occur through the use of the technology (Lai, 2008). In information technology, the Technology Acceptance Model (TAM) posits the intention to use a technology is positively related to the perceived usefulness and the ease of use (Davis, 1989). Recognizing the variability of psychological and sociological traits, researchers have extended TAM to include external variables including social influence and cognitive processes (Venkatesh and Davis, 1996). Other researchers have found a positive relationship between self-efficacy and online learning (Grandon et al., 2005; Park, 2009) and although some found an mediated effect of self-efficacy through intention to use (Grandon et al., 2005), Park (2009) found that self-efficacy was the most significant variable in predicting intention to use. However, children may not behave in the same way and are more impacted by the technology they are accustomed to and the adults in the environment; however, a number of factors can affect these results, including level of experience with technology, parental attitudes, and access to technology (Pruet et al., 2016).

The interaction between students and teachers has been shown to increase students' performance in learning. Furthermore, teachers' self-efficacy can be related to students' performance and ability (Corkett et al., 2011). Teacher's self-efficacy with technology, therefore, can become a major contributor toward the successful adoption of any type of online learning, including artificial intelligence enabled learning. Even though AI should be autonomous, the student will most certainly look to a teacher or an adult for positive reinforcement. Thus, not only should positive reinforcement come from the technology itself, but also from the trusted adults around the learner. As teachers use technology more, they become more familiar and comfortable with technology, thus usage is a significant predictor of self-efficacy (Albion, 2001). Positive attitudes toward the technology will not only increase the usage (Herman, 2002) but improve the outcomes in classroom and online learning environments (Delcourt and Kinzie, 1993; Herman, 2002).

Student learning and approaches to learning are very different, and artificial intelligence and online learning do not necessarily need to universally affect students negatively. In online environments, student outcomes have varied based on a number of other factors. Research has shown that students who struggled with executive functioning skills like task initiation found their struggles amplified in an online environment. However, students who struggled in school due to organizational skills improved during remote learning on account of the way their work was organized on online platforms for them (El Mansour and Mupinga, 2007).

Any technology application, regardless of its synchronous or asynchronous application, possesses a number of dynamics, which any artificial intelligence technology would need to address. Understanding and analyzing these dynamics will help a technology solution dynamically adapt to student needs with as minimal human interaction as possible. The literature demonstrates that providing a level of feedback and support aligned with the best approach for the student will likely have the most positive outcomes.

### Artificial intelligence in education

Earlier work in Decision Support Systems and Knowledge Support Systems represent the foundation of using technology in education. The exploration of AI in education can be seen with the creation of the Journal of Artificial Intelligence in Education in 1989. Subsequent articles have explored technological attributes such as Betty's Brain, Intelligent Tutoring Systems, AI Infrastructures, etc. (Leelawong and Biswas, 2008; Holstein et al., 2018; Williamson and Eynon, 2020). The research shows a broad set of technologies and applications geared toward the goal of learning. As we move toward AI as a teacher in education, it is necessary to explore specific research that focuses on the interaction between teachers and students, especially at a K-12 level.

Leelawong and Biswas (2008) studied the results on student learning through a computer-based, teachable agent called Betty's Brain. The study extends work from Gonzalez and Kasper (1997), which posited that increased interactivity in learning has a positive effect on decision making, as well as findings from Mullikin (2020) that children need feedback in the form of comments. The interaction between Betty and the student instructor was the focal point. Since Betty directly provided feedback to the student instructor regarding her mastery on a topic, this feedback allowed the student instructor to adjust their knowledge in order to teach Betty to succeed on the quizzes. These self-regulated features in Betty assisted the student in their preparation in order to help Betty (Leelawong and Biswas, 2008). Findings revealed that those students learning the subject through Betty, the teachable agent, showed higher learning gains than those students who used a traditional ITS system. The study also highlighted the importance of self-regulated learning and how it transfers to future learning for students, showing that those using the self-regulated form of Betty spent more time reading resources to understand a subject in new domains (Leelawong and Biswas, 2008). Although the study demonstrated increased learning and highlighted the importance of self-regulating behavior, more research is needed to explore these issues of even younger grades and different outcomes in qualitative topics such as history and English.

Beyond Betty's Brain, ITS research continues to expand as more robust analytics are integrated with increased and more immediate feedback to explore more positive outcomes in learning. Holstein et al. (2018) examined the effects of student learning when combining an intelligent tutoring system (ITS) with teacher monitoring that has been enhanced by real-time analytics using a pair of smart-glasses called Lumilo, alerting teachers to real-time deficiencies students were experiencing during their interaction with the ITS tool. They found that among the students taught by the teachers using Lumilo, the enhanced monitoring by the teacher had a positive impact on student learning compared to those taught by teachers without the real-time analytics information (Holstein et al., 2018). The study noted the positive effects of AI Education (AIED) systems combined with human learning. Building upon literature of this type can help explore aspects of where AIED may fall short in the learning process, specifically around socialization and other psychological issues students face in a K-12 environment.

ITS tools provide ongoing feedback to students and create a dynamic challenge response mechanism with increasing difficulty as students' progress (Holstein et al., 2018). However, the ITS tool itself does not adjust the teaching method for the student and employs the same method for each student, one that may be helpful for one group of students but not another. Therefore, traditional ITS tools may lack the diversity and adaptability to adjust to individual needs.

Holstein et al. (2018) utilized real-time analytics along with an ITS in an attempt to close the learning gap between those students with higher and lower learning ability by alerting the teachers to student deficiencies in real-time. However, the author's noted that teacher experience may be a critical factor. Previous research would highlight the need for teaching self-efficacy and technology self-efficacy as possibly being antecedents to effective use of ITS. This highlights an important question as to the sustainability of this type of teaching/learning environment. What would be the results of learning in a large classroom where multiple students were having trouble with the same lesson? An AI tool should be highly responsive to individual students' needs and challenges, with only minimal and necessary interaction of a teacher. Developmentally, children at younger ages are learning not only academically, but through social cues and social modeling of their peers and teachers, so it is critical that students feel an emotional or social connection to the technology, i.e., it must feel as though it is an extension of the teacher, with whom the students have a connection and trust. Understanding the social implications and emotional impacts has not received sufficient attention from researchers (Karnouskos, 2022). Intrinsic factors such as sociability, enjoyment and adaptability have been shown to be key antecedents of AI acceptance, specifically robots (De Graaf and Allouch, 2013). The behavior and outcomes of students would be impacted by the interaction, attitudes and emotions toward the technology (Karnouskos, 2022). This concept is being employed by companies researching “emotional robots” that serve in the same capacity as support animals for therapeutic purposes (Karnouskos, 2022).

## The Turing Teacher

Earlier, it was noted that Alan Turing provided a test for Artificial intelligence, i.e., when a human cannot distinguish its interaction between another human, e.g., teacher, and an artificial intelligence technology. The Turing Teacher, therefore, is a piece of AI technology in which the student cannot distinguish between a real teacher and a simulated interactive teacher, such that the learning outcome and satisfaction with lessons would be equivalent.

By exploring the online learning environment, the use of artificial intelligence in education can be more successfully targeted and applied. Examining the experience of teachers, parents and students, by proxy of their parents, can shed light on what would be needed by a Turing Teacher. While adults are more accustomed to technology and are more able to distinguish technology from a human, we posit that the Turing Teacher is a piece of technology in which a student's learning behavior is indiscernible from one in which the human teacher is physically present. While the measure of success would include traditional measures, such as test scores, positive outcomes would need to also assess satisfaction, creative thinking, executive functioning as well as soft skills, including ethical attributes, i.e., students behavior is aligned with expectations from a classroom environment.

In order to develop a set of characteristics for a Turing Teacher, data must be collected from a broad range of areas, psychology, education, business, and technology. It is important to recognize that each educational topic is different, and so there cannot be a one-size fits all approach. Furthermore, since students learn and behave differently, any AI technology must be able to adapt to these dynamics to gain the desired qualitative and quantitative outcomes. The complexity of the information in an educational environment requires that AI technology rapidly adapt to the changing needs of educators to help mitigate any cognitive limitations (Samuel et al., 2022). By exploring the challenges of an online environment, specifically, one that was not planned, i.e., an online environment created to mitigate the issues caused by the pandemic, we get a view of the immediate challenges from the perspective of the key stakeholders. The study was geared to collect quantitative and qualitative information to assess how well the expectations aligned with the literature review.

Using a study conducted in online learning, we aim to develop a set of core attributes that would be needed to help create a “Turing Teacher” test. Our attempt, therefore, is to establish what is required so that a K-12 student would, at the very least, not be able to distinguish between learning from a human teacher and learning from a technology, or AI source. The study was a short longitudinal study during the height of the COVID-19 pandemic, and followed the teachers' progression in the online setting as well as parent perceptions at the beginning of the pandemic and at the end of the school year. We explore the challenges and concerns parents and teachers have in order to build the foundations of the core attributes of AI in K-12 education.

## Methods

### Research design

This research was a mixed methods case study of a small private school on Long Island, New York. The school serves approximately 120 students in grades K-8 and has 17 teachers. The school has been in existence for over 30 years and its educational model focuses more on experiential learning rather than test based learning. The school serves as a pilot school for a program at a nearby university in which students from the local university teach technology education such as Python, Excel, SQL and Scratch. The program had plans in place to move to a hybrid online learning environment with synchronous and asynchronous learning modules. However, this portion of the program was only at the conceptual stage when the pandemic and subsequent closures hit.

The COVID pandemic lockdown created many issues for the school, as there was no clear and consistent guidance from state and local governments, and as such, the school was forced to create a plan for educating the students. Using the initial plans from the university based program, the school pivoted quickly and provided synchronous and asynchronous online learning through the implementation of Zoom for Video Conferencing and a learning management system called Canvas. The director of the university program acted as the technical lead and coordinator since the director had executive level technology experience for large corporations.

Simultaneously, using previous research, a quick survey based in education and information systems literature was created. It was decided to produce a survey at the beginning of the online instruction for teachers to identify their feelings and attitudes around online education, and its impact on students, as well as a survey for the parents to assess their feelings of online education. Each week teachers were asked to complete a survey based on their experiences for the week, which would be used to assess changes in feelings and attitudes of their online instruction as well as gather intelligence on how the teachers were adapting to the technology. Finally, when the school year completed about 12 weeks later, a final survey of teachers and parents was conducted to see how their attitudes may have changed. Each survey had both quantitative and qualitative responses.

Due to the limitations i.e., size of the school, speed at which the survey needed to be created and communicated as well as simultaneously dealing with the pandemic, data collection became a challenge; however, we believe the results are valid in understanding the challenges of the online environment and can serve as a springboard for artificial intelligence in education.

## Results

### Teacher results

Teachers were asked the same set of questions at the beginning of the lockdown and then three months later when school ended. The literature review discussed a number of items related to online education including self-efficacy and teacher attitudes toward technology and their impact on the perceived use of technology and thus the use of technology effectively.

Thirteen teachers responded to a pre-transition survey at the onset of the school closure, and 15 teachers completed the post transition survey at the end of the semester. In addition, weekly surveys were conducted to gauge teachers' changes in experiences over the semester. School closure lasted from mid-March until the end of the school year in mid-June. The survey asked participants about their attitudes toward technology and online teaching, as well as their perspectives on the usefulness and ease of use of the Learning Management System (LMS) as prescribed by the Technology Acceptance Model (TAM) theory. Open text responses were used to gauge the effectiveness of the technology in teaching. Table 1 represents the mean scores of both the pre and post surveys. All items were measured using a 7-point Likert Scale, with 1 being strongly disagree and 7 being strongly agree.

**Table 1:** Mean scores on pre and post attitudinal surveys.

	**Items** **(Likert 1–7)**	**Range of points**	**Pre** **(*N* = 13)**	**Post** **(*N* = 15)**
Attitudes toward technology	3	Min: 06	14.75	15.53
		Max: 20		
Attitudes toward online teaching	7	Min: 16	28.00	30.87
		Max: 44		
Attitudes toward LMS usefulness	6	Min: 10	24.75	26.80
		Max: 42		
Ease of use of LMS	6	Min: 10	27.50	27.47
		Max: 42		
Confidence in online teaching	9	Min: 21	38.92	43.60
		Max: 58		

Attitudes toward technology were measured with three items. The mean attitude score prior to the transition was 14.75. Attitudes among all participants but one were high and positive. Attitudes toward online teaching were unsurprisingly low to moderate. Similarly, scores were low to moderate for the perceptions of usefulness and for ease of use, likely because teachers had no experience as to the use of an application like Canvas. In both groupings, a favorable score would be 30 or higher.

Surprisingly, with respect to confidence in teaching online, participants reported relatively moderate perceptions of their abilities, despite teachers having little experience with online teaching. About half of the participants rated each item as agree or strongly agree while the other half did not agree with statements regarding confidence in their impact with online learning. As expected, after the transition, teachers became slightly more confident, likely due to increased experience. Conversely, the gains may have been impacted by increased teacher frustration over a period of online teaching that went on far longer than anyone anticipated. In addition, the frustration may have been fueled by a lack of student participation and technology problems.

Using the multivariate Hotelling's T^2^ test, we examined the data to see if there were any differences between the start and end of the survey, however, no differences were found to be significant. There are two possible reasons for this. First, because the school is small with 18 teachers, the small sample size could be a limiting factor. We did analyze the individual items and also no statistically differences between any of the items as well. We further posit that the stress placed on the teachers may have been a factor in their responses. While the data showed no differences, it was important for this research to provide these results stressing the difficulties of an online teaching environment and that even with some experience, favorability didn't change.

The survey provided space for weekly comments by the teachers. Using this freeform text we used popular sentiment libraries in R to assess positive and negative emotions. Sentiment analysis has been used to analyze twitter messages, online comments, and areas such as conservation science and finance (Valdivia et al., 2017; Lennox et al., 2020; Kausar et al., 2021; Qian et al., 2022). Using sentiment analysis for small responses such as twitter messages has specifically been used to examine pandemic responses and sentiments toward opening up the economy (Samuel et al., 2020a,b; Ali et al., 2021; Rahman et al., 2021). These authors also advocate for more usage of sentiment analysis techniques in research since the responses can reveal rich insights. Thus, we examined the weekly responses of the teachers to see if we could find any indication of emotion as the school year progressed. Using the Bing NLP library in R, we generally found a slightly negative emotion in their responses as the weeks progressed (ϱ = −0.20) however, the *p* value was not significant at the 0.05 level (*p* = 0.07, *n* = 78). While the overall sentiment analysis was inconclusive, we further explored the text from the beginning of the study and the end of the study which does provide some interesting insights. We excluded from further analysis the weekly comments, since the focus of the weekly comments were dedicated to any comments the teachers wished to provide about the week of teaching, whereas the beginning and ending comments asked about thoughts about online education. In addition, since the same question was asked to the parents it provided the best comparison for both teachers and parents attitudes toward online learning.

### Results from teacher text responses

At the beginning and end of the survey, a single question was asked, “Please share any thoughts you have about online education, in terms of effect on children, quality of education, challenges to teaching, etc.” We used a deductive coding method (Azungah, 2018) from our literature review to provide a foundation for assessing the comments of teachers. We deliberately created codes that were technology agnostic to focus on the participants' attitudes about online teaching (**Table 3**). The codes used for teachers were Oversight, Emotion, Communication, and Self-Efficacy. Oversight was defined as any text that referenced control of the classroom, methodology, executive functioning of students, or changes to lectures due to the online education. Emotion was classified when the responses were highly emotional or expressed emotion of the students. When the respondent focused on the mean, frequency or quality of the interaction with students, these responses were classified as Communication. Finally, if the responses focused on either their ability or the ability of the students to function properly within the online environment, the responses were classified as Self-Efficacy.

The responses for the teacher comments from the start and ending survey (*n* = 20) were given to two independent coders along with the classifications obtained from the literature review. Once the coders categorized the responses we tabulated the inter-rater reliability measure using Cohen's Kappa. Our analysis showed very substantial reliability (Davies et al., 2018) between the two coders for the teachers responses κ = 0.625.

From an oversight perspective, the coders found that about 20% of the responses dealt with some form of control over the classroom or how the students learned. Although most teachers expressed concerns about screen time and the inability to have control over what students did with their learning, most expressed that online learning can be useful and a “lifesaver” in some instances.

“*It is a valuable tool that allows teachers more freedom and the ability to reach more students. It was positive for students who may need to be home for illness or other reasons, particularly when students return to in person instruction in the future.”*

“*Overall, the kids were able to adjust. But not all of them. The education took place but it could not replace the classroom setting… We could all use training to be alert to the signs of student behavior that would trigger a need for more attention.”*

“*My sense was it was easier for them to not pay attention during class, participation worsened greatly, and many students got by with the least amount of effort. My sense is gamification is likely more important with online learning than it is with in-person learning.”*

Surprisingly, only two of the responses were coded as emotional. The teachers stressed the need for the emotional connection to their students and indicated that they did not want to teach this method or that this type of learning was suitable for older students.

“*Online learning feels too mechanical, lacking warmth despite kindness, and kind words being spoken. Ultimately, online potentially fine for older students.”*

“*The contact was there but not the eye or emotional contact. We did our best but I don't want to continue teaching this way.”*

We found similar to the oversight classification, communication coded responses were detailed around the interaction between the student and teacher and varied anecdotally by grade. Much of the concern around communication centered on the technology itself, availability or training, but in general the comments presented did not seem as though the challenges were insurmountable.

“*Just not 100 percent of the class handed in their work. Maybe 85 percent did all assignments perhaps? Reasons varied student to student… Lesson planning took a lot of time and was hard to keep up with,... I got excited to see the assignments come in on canvas, and to see students, laugh and understand lessons on zoom. I often asked for photo downloads of work and pictures. It helped to really see how the students were connecting with the material and projects.”*

“*Online education is a valuable tool. It allows teachers more freedom and the ability to reach more students. It is a positive for students who may need to be home for illness or other reasons.”*

Self-efficacy comments were the highest, with 40% of the responses being coded as dealing with capabilities of technology or teaching online. In some cases, teachers tried various methods to help overcome challenges; however, other instances demonstrated a clear lack of efficacy in teaching online.

“*I would say the biggest challenge for me was to convert the traditional methods of teaching into ways to keep students engaged and productive… I used educational sites, interactive game sites, and youtube videos. I found that for me, I could not just transfer what I had been doing to online learning in many ways, and it was very time consuming to reform my lessons to suit their needs online. But I did it because I wanted them to stay invested in school, and to help take their minds off of the stress and worry that many families were facing.”*

“*I cannot teach my students in real time [online].”*

“*For eighth grade, canvas was incredible. The kids didn't fall off at all and either continued their success or did better. Seventh grade saw a fall off with their performance, having not been with my learning and teaching style for a full year prior to this. I think next year they will be better if we begin the year this way, but I fear for the incoming 7th grade. I loved Canvas and hope to use it further with in class learning for assignments as well.”*

The statements above, across the four categories demonstrate the challenges that an artificial intelligence tool will face toward greater adoption. The acceptance of online learning or a technological learning tool is not seen as a possible replacement but rather a possible compliment or temporary substitute. The ability to provide synchronous learning and teaching is essential to humanizing online learning, particularly with elementary aged children. As one teacher reported,

“*Connecting with the class during zoom meetings serves a meaningful purpose where lessons are possible in a group.”*

Teachers indicated that online learning cannot be simply an unattended technology but it must somehow be complementary to the teacher not just a “set it and forget it endeavor.” Rich media technologies can help with connection to the students since the primary task of teaching is conveyance, and thus online conferences *via* zoom can “help humanize and be a place for motivational talks possibly that can be followed up with [email messages].”

The synchronous component is critical for learning both in an individual and group setting. As the literature review indicated (Dignum, 2021), interactivity is one of the key components for AI tools. However, this interactivity cannot simply be a question/response type of interaction. The humanizing effect that teachers indicated means that the AI tool must be able to get responses from the students, assess the mood and emotional state of a student and provide appropriate feedback, both quantitatively and qualitatively, to the student. This response loop should yield better self-efficacy both from a student perspective and teacher perspective.

Teachers noted the lack of executive functioning such as ability to keep students on task and manage their time as an overall function of oversight. Executive functioning is a group of processes reliant on a student's cognitive capability in interactions allowing a person to act in a deliberate manner (Gioia et al., 2000). As part of classroom management, teachers help students with their executive functioning which is lacking in an asynchronous teaching environment and a virtual synchronous teaching environment. The learning management systems allowed the teachers to hold students accountable for dates and times; however, real time guidance was not possible. In the same way AI might fly a drone, in which constant corrections are being made for weather variables, an AI education tool must learn to help students make minor corrections as an assignment is being done or as a lesson is being conducted. Teachers reported that although independent work was submitted on time, there were ”[more] errors and working collaboratively was not possible.“ In order to mitigate the issues, teachers would provide one on one video conferences with students, with some teachers doing over 20 in a week. One teacher noted that while children's attention may wane during video conferences, their solution was to hold synchronous sessions and…

“*...use a Wacom tablet, which proved effective in allowing children to follow along while the teacher did the math problems.”*

Although emotion did not appear as much as other topics, teachers did express some concerns about young children and screen time. As one respondent noted,

“*Just as email cannot pick up emotion and inflection, on-line teaching is “cold”.”*

A Turing Teacher would need to be viewed in a manner that is “not cold.” While using technologies such as Natural Language Processing and facial recognition might help, it is clear that human interaction is critical. Finding a suitable way to substitute this interaction is important; however, the answer may be as simple as having an AI include a real teacher when certain outcomes are not possible within the AI technology, or having as close to a real simulated teacher as possible with recorded videos, changing facial expressions and voice tonality.

The situation may be analogous to a younger teacher, in their first job, or as a student-teacher, where the teacher does not have a full set of experiences in teaching, or is unable to reach a student. In this case, the teacher uses the means they have been provided but may seek guidance from a more experienced teacher or one with a unique set of skills. The Turing Teacher must be able to assess when it is not achieving the required quantitative and qualitative outcome and have means by which a more experienced human teacher can be of assistance.

### Parent results

Parents were also asked the same set of questions at the beginning of the lockdown and then 3 months later when school ended. Fifty-one parents responded to the pre-transition survey, and 53 parents completed the post transition survey at the end of the semester. The survey focused on attitudes toward online learning. Open text responses were also used to gauge parents' responses. Table 2 represents the mean scores of both the pre and post surveys. All items measured were on a 7 point Likert scale (1—strongly disagree to 7—strongly agree).

**Table 2:** Mean scores on pre and post attitudinal surveys.

	**Items**	**Range of points**	**Pre** **(*N* = 51)**	**Post** **(*N* = 53)**
Attitudes toward technology	7	Min: 7 Max: 47	29.41	24.08

We used the multivariate Hotelling's T^2^ to examine the effect of all 7 items simultaneously against the pre-test and post-test group. The results showed a statistically significant decline in the attitude toward online learning from the pre-test to the post-test (T^2^ = 5.76, *p* < 0.01). We caution the reader on the result however. While the test was significant, the attitudes of many parents may have been biased due to attitudes around the pandemic, and while the negative attitudes toward online learning are apparent, it isn't clear whether this was simply a result of pandemic frustration and anger.

### Results from parent text responses

From the literature, we established four classifications similar to those used for the teachers (Table 3). The codes used were Oversight, Participation, Learning Environment, and Self-Efficacy. Oversight and Self-Efficacy were defined from the literature in the same way they were defined for the teachers free form text. Participation was the classification used since parents could gauge whether or not their children were engaged with the learning, lessons or group. Learning Environment was a broader classification, which was used to identify whether the parents believed that the online learning, or technological instruction was conducive to positive and quality learning.

**Table 3:** Classification table of core attributes.

**Teachers**	**Students**	**Parents**
Technology self-efficacy	Technology self-efficacy	Technology self-efficacy
Control/Oversight	Executive functioning	Oversight capabilities
Emotional recognition	Motivation	Level of participation
Communication	Socialization	Learning environment

We were able to use 61 responses for the parent comments and these responses were given to two independent coders along with the classifications listed above. Once the coders categorized the responses, we tabulated the inter-rater reliability measure using Cohen's Kappa. Our analysis showed moderate to substantial reliability (Davies et al., 2018) between the two coders for the parent responses (κ = 0.585).

The results from the independent coders showed a strong focus on Learning Environment and Self-Efficacy. Oversight and participation, collectively, received between 15 and 20% of the classified responses of the two coders. Oversight responses generally focused on the shifted responsibility of monitoring the children from the teacher to the parent.

“*Online instruction would probably work better for middle school students and possibly very motivated late elementary schools students. For younger students and reluctant learners there will be a great burden on the parents.”*

“*It is very hard to work full time and home school a young child.”*

Participation responses also seemed tied to parent capabilities or children's executive functioning at home due to distractions or a lack of guidance.

“*As the period of online learning progressed, both my child and the school staff seemed to hit their stride. It was bumpy and frustrating at the beginning. Both my child and I were often confused about where to find things on Canvas and knowing when things were due. My son had difficulty staying on top of assignments. …We had to implement a pretty strict schedule at home in order to keep him on task.”*

“*Students may not be able to complete their assignments if their parents are working full time and there are multiple children at home.”*

“*...she only saw her teachers 2 to 3 days during the week. So it came across as a 5 day weekend and 2 days of school. Needed more interaction between student and teacher. assignments being given becomes like homework.”*

The learning environment and self-efficacy clearly weighed on the parents. Obviously, the pandemic created certain undue stresses, however, the parents noted that for some classes the online learning worked well, but that ultimately these solutions should be temporary or complimentary. The first comment below highlighted the difference in outcome for a language class but didn't feel the same for a more quantitative class.

“*Online learning works well for Spanish class, but not for Math and Science which needs more interaction.”*

“*Online school was good as a temporary solution during quarantine.”*

Some parents did recognize a big challenge in teaching in the learning environment by stating “children cannot learn the same way as in person.” The learning issue further provided evidence regarding the executive functioning aspect of learning, with a parent stating.

“*My child did not enjoy virtual learning at all getting them to focus was an extremely difficult task. They were not motivated to do the work.”*

It became evident that being able to stay on task was a necessary function and the disconnect between the teacher and student due to the technology was a critical factor. In an AI setting, the technology must be able to help the student stay on task in an interactive and positive way and thus have a positive impact in the learning environment.

The stress of online learning and the lack of connection with their peers was also mentioned a few times and could be a key factor in the negative attitudes toward online learning. One parent indicated “online learning was stressful for the children and parents.” The coders noted these issues as a learning environment issue. The stress could be attributed to a number of factors including pandemic related issues, providing assistance to children to stay on task and learning a new technology to guide the children.

Parents were required to learn about the technology to help their students join classes or use the tools. This uncertainty in technology increased the stress for both parents and students. One response indicated that “Children need to learn, but they also need in person socialization.” In a classroom environment, students are able to rely on their peers, and alleviate some stress through their social connections. The AI tool cannot provide in person socialization, but the social interaction might be facilitated by creating small dynamic breakout rooms or having pictures of at least two of their peers on a screen, such as a left and right peer.

Parents were also not of the belief that younger students would benefit from the technology or be able to handle the learning environment. In addition, there was a belief that the technology was harmful to the children, with responses such as:

“*Online education should be left to college students and beyond.”*

“*Expecting children to sit in front of screens all day is unhealthy in so many ways.”*

Another major concern for parents was whether they had the skills or knowledge to navigate the technical challenges or whether they had the aptitude to handle technical challenges. In some cases parents who had stronger capabilities could provide more support and were able to navigate the obstacles.

“*Children adopted to online learning and tried their best to keep up with assignments. Technology part was the easiest.”*

“*Considering the fact that the children had no time to transition or preparations from the teachers, I think most of them did extremely well (if they had supportive parents). Without parental support, many of the kids gave up completely and threw the whole idea away.”*

“*My son really enjoyed the online education via Zoom & Canvas. He had no trouble adjusting from going into an actual school building to doing everything through a tablet. Parents and students should be given ample time to transition to online learning.”*

“*Online education can be useful if students have been prepared prior to using the internet and the web as a learning tool. There is a learning curve and a shift that kids will have to make initially that can be frustrating if it is all thrusted upon them at once. The computer is no longer just for entertainment and fun games but instruction like school.”*

Parents as observers of their children in the online setting, provided similar feedback to the teachers, although their perspectives changed for the worse from the beginning of the initiative to the end. They cited stress, lack of knowledge of the technology, lack of executive functioning and a disdain for the overall learning environment, in many cases as being the main reasons for a negative outcome.

## Discussion

As AI technologies develop the need for deploying them in an education setting will continue to grow. The results above present the challenges faced by any technology in an education setting. Technologists must resist the temptation to deploy AI technologies that are simply question/response focused. Even highly adaptive technologies that can learn from student responses will still be limited in their effectiveness as an AI teacher. From our results, we develop the foundation and ultimately a test for the concept of a Turing Teacher.

We found that although there might be support by the teachers for online technology implementation was considered “limited” in both application and scope. We further found that parents became more negative as time progressed and voiced their frustrations in the open ended text. Teachers and parents are very good proxies for the progress of the children in classes and thus, the value of their responses cannot be understated. These adults can judge the qualitative attributes of the education such as motivation, efficacy and satisfaction better than any measurement of outcomes.

The Turing Teacher evolves from Alan Turing's test for artificial intelligence; however, we provide some specifics around the characteristics of the Turing Teacher, beyond the defined AI attributes of autonomy, adaptability and interactivity. From the qualitative responses, we derive some additional attributes for each stakeholder, teacher, student and parent. The attributes help identify a theoretical foundation to aid in developing paradigms for a Turing Teacher and online education. The intersection of the associated skills between teachers, students and parents, coupled with their own self-efficacy in technology are critical components. From the qualitative responses of teachers and parents therefore, attributes for students were derived and shown in Table 3.

In order to achieve success, the Turing Teacher must address each of these attributes for its stakeholders in a way that is meaningful and leads to positive outcomes. First, just as a student must learn to adapt to a school or classroom environment, the Turing Teacher must help the student adapt to its environment. The technology needs to adapt and help students navigate the material and technology. This will help promote self-efficacy in the technology for the student, but simultaneously for the teacher and parents.

Second, the Turing Teacher must enable oversight functions for a human teacher and parent. The adaptive capabilities of the technology can help identify students that are falling back in material such as math problems, but the technology must go further. The technology must be able to sense changes in participation, quality of participation and make changes to help increase both or advise a human teacher of the issues. Similarly, the Turing Teacher must help students who have difficulty with executive functioning. As part of the oversight the tools developed need to assess how students are participating and develop or use different means to help students improve the executive functioning.

Third, the Turing Teacher must help students in group settings and group projects. Identifying how students collaborate *via* text, audio, video or in person, the information needs to be assessed by the Turing Teacher to identify how students are operating in a group setting a critical component of a student's learning. When students are not successful in group settings the Turing Teacher should be able to identify means to help the student improve in the engagement or alert a human teacher as to the issues.

We view the Turing Teacher as a teacher whose functions should be the same as a regular teacher. It does not mean the Turing Teacher must be successful at all of the activities, but it must be able to operate at some level of all of these types of activities and seek assistance from a more “experienced” human teacher.

## Conclusion

We attempted in this paper to leverage an opportunity presented by the pandemic. The pandemic forced schools to go to technology driven education. Using information gathered from a pilot program in a school that had some basic technology but was not equipped or trained in a specific technology gave us a unique opportunity to see the raw responses of teachers, students and parents simultaneously. From this information we could see the challenges faced and leveraged research to develop the concept of a “Turing Teacher.” A Turing Teacher would need to overcome the challenges faced by students as reported by teachers and parents. Specifically, the technology must be motivating and help foster a connection between the student and the teacher. Future research should be focused on examining the intricate psychological and sociological relationship between the AI technology and outcomes for teachers and students. Holstein et al. (2018) argued for more work to understand the complex interaction and explore the “complementary strengths of human and automated instruction.” Effective implementation Human AI interaction can lead to benefits in education enabling different learning styles and needs of students (Holstein et al., 2018; Miller, 2019). Thus, we recommend that future work in AI for K-12 education focus on the attributes of the Turing Teacher including executive functioning, motivational and socialization issues for students. Simultaneously, the technology must adapt to the needs of teachers, students and parents as well, who are the overseers of their children's education, in the same way teachers interact with parents in an in person setting. Overall the tool must foster a belief in better outcomes and processes such that the technology must not be its own obstacle. Thus, an intuitive and simple to use interface that can provide not only the lessons but meet the soft skill requirements are key attributes of the Turing Teacher.

The positive impact of a Turing Teacher is enormous. Turing Teachers could help alleviate issues with the teacher shortage in the US. Further, the technology could be implemented to help students who are in need of special services or who may not have access to quality education, potentially closing the gap in education seen in areas of poverty. Turing Teacher systems could inherently remove bias from attention provided by teachers to some students or even grading. Students could be served better in some classes based on their needs and thus relieve pressure from human teachers, whose time could be best utilized elsewhere. Overall, including these core attributes can help the research progress and lead to better overall outcomes in K-12 education.

## Data availability statement

The raw data supporting the conclusions of this article will be made available by the authors, without undue reservation.

## Ethics statement

The studies involving human participants were reviewed and approved by Hofstra University IRB. Written informed consent for participation was not required for this study in accordance with the national legislation and the institutional requirements.

## Author contributions

All authors listed have made a substantial, direct, and intellectual contribution to the work and approved it for publication.
